# Torpor-Induced Regulation of Poly(A) Tail Machinery in 13-Lined Ground Squirrel Brown Adipose Tissue

**DOI:** 10.3390/jdb14020021

**Published:** 2026-05-14

**Authors:** Saif Rehman, William G. Willmore, Kenneth B. Storey

**Affiliations:** Department of Biology, Carleton University, Ottawa, ON K1S 5B6, Canada; billwillmore@cunet.carleton.ca (W.G.W.);

**Keywords:** metabolic rate depression, torpor, epigenetics, poly(A) tail, deadenylation, *Ictidomys tridecemlineatus* (13-lined ground squirrel), mRNA, transcription

## Abstract

The poly(A) tail has long been known to play a central role in mRNA stability, storage, and translational competence, making it a potential key regulator during hypometabolic states. During seasonal torpor, hibernating mammals must frequently enter these hypometabolic states to survive. In this study, we examined protein abundance changes in key enzymes involved in poly(A) tail synthesis, binding, and removal during torpor in the brown adipose tissue of the 13-lined ground squirrel, *Ictidomys tridecemlineatus*, using immunoblots. BAT during late torpor exhibited significantly reduced abundance of the catalytic cleavage enzyme CPSF73, but increased abundance of poly(A) polymerase PAPOLA. In contrast, poly(A)-binding proteins and major complex subunits of deadenylases, including CCR4-Not, exhibited no significant changes. Furthermore, despite unchanged levels of the translation initiation factor eIF4E, the phosphorylated variant of 4E-BP1, a potent inhibitor of the initiation factor when hypophosphorylated, was significantly reduced during late torpor. Overall, constrained mRNA maturation, preserved transcript stability, and reversible translational inhibition suggest that an important role exists for poly(A) tail regulatory machinery in hypometabolic survival throughout the torpid state.

## 1. Introduction

Strategies for surviving prolonged periods of cold with limited resource availability range across taxa, with some mammals adapting hibernation as their primary method. The 13-lined ground squirrel, *Ictidomys tridecemlineatus*, utilizes a controlled entry into torpor undergoing a drastic reduction in body temperature from euthermic temperatures down to ambient levels of 0–5 °C. This significant decline is coupled with metabolic rate depression (MRD), where the metabolism reaches basal levels near 2–4% of normal activity [1,2]. Torpor is also characterized by marked suppression of respiration and blood circulation, analogous to hypothermic and ischemic conditions [3,4]. Despite these stresses, ground squirrel tissue remains viable and can rapidly resume normal functions through bouts of arousal through the torpor period [5,6]. The ability of the tissue to go from a quiescent state to full activity and metabolism makes it an ideal model for molecular adaptation study, specifically for further understanding long-term survival under hypometabolic conditions. Past studies have specified brown adipose tissue (BAT) as vital to successful arousal, and thus survival across torpor–arousal cycles [7]. Given its role in engaging non-shivering thermogenesis to induce arousal, brown adipose tissue experiences extreme metabolic changes throughout torpor cycles. As BAT is among the first tissues activated prior to arousal and requires a rapid return to function, it serves as an outstanding model for demonstrating any significant regulation required throughout the stress.

Prolonged bouts of torpor require extensive cellular reprogramming, in large part to preserve ATP for metabolic reactivation. Entrance into a torpid state influences the activation of MRD, resulting in energy-expensive processes such as transcription, translation, and protein turnover being strongly suppressed [8,9]. Gene expression is often not completely halted during MRD as pathways essential to tissue survival such as anti-oxidant defense and anti-apoptotic signaling must continue to function, whilst minimizing ATP expenditure where possible [10,11]. Cells transitioning into a quiescent or differentiated state similarly require a period of suppressed proliferation without loss of viability [12]. Post-transcriptional regulation seems to play a vital role in both systems by imparting regulatory control over transcripts in a rapidly reversible manner.

Poly(A) tail regulation appears as a determinant factor in post-transcriptional mRNA’s fate. The poly(A) tail is a stretch of adenosine residues that are added to the 3′ end of eukaryotic mRNA by a poly(A) adenylase (PAPOLA) following cleavage of the pre-mRNA by the cleave and polyadenylation specificity factor (CPSF) [13]. The poly(A) tail length strongly influences mRNA’s fate through interactions with poly(A)-binding proteins such as poly(A)-binding protein nuclear 1 (PABPN1) and poly(A)-binding protein cytoplasm 1 (PABPC1) [14]. Initiation factors, such as eukaryotic initiation factor 4E (eIF4E), which acts as the rate-limiting step in recruiting the 40S ribosomal subunit, rely on these binding protein interactions to bind to mRNA transcripts and prime them for translation [15,16]. The action of poly(A) tail deadenylases such as CCR4-Not complex proteins, along with PAN2-PAN3, PARN, ANGEL2, and NOCT, shortens the poly(A) tail length to further exert regulatory control over poly(A) tail dynamics [17,18,19,20,21]. Strict and intentional control of these processes allows cells to regulate mRNA turnover and translational competence without committing to the production of entirely new transcripts, which would be energy-inefficient in a hypometabolic state.

Regulation of poly(A) tail machinery could prove an efficient mechanism of gene expression regulation, especially in a state of torpor that would require strict gene expression control. The present study investigates changes in the protein levels of key enzymes involved in poly(A) tail synthesis, binding, and deadenylation in 13-lined ground squirrel BAT during late torpor. By better understanding how the poly(A) tail is regulated during torpor, this work sheds light on the effect of post-transcriptional machinery on a reversible hypometabolic state.

## 2. Materials and Methods

### 2.1. Animal Treatment

Male ground squirrels (*Ictidomys tridecemlineatus*) weighing 150–300 g were captured and transported to the Animal Hibernation Facility, National Institute of Neurological Disorders and Stroke (NINDS) (NIH, Bethesda, MD, USA). All hibernation experimentation was conducted here. The NINDS animal care and use committee (ACUC, #ASP 1223–05) approved both animal house and experimental procedures. Select 13-lined ground squirrels were housed individually in a shoebox cage at 21 °C. Animals with sufficient lipid stores to enter hibernation that weighed between 220 and 240 g were randomly allocated to control or hibernation groups before being placed in their respective environments. The control ground squirrels were housed in a cold room and had a Tb of 37 °C at the time of euthanasia. To induce a natural transition into torpor, animals were transferred to an environmental chamber at 5 °C in constant darkness. Body temperature (Tb), time, and respiration rate were observed to determine sampling points, as previously described [22,23]. The late torpor stress condition refers to animals that were continuously in deep torpor for at least 5 days, with Tb values of 5–8 °C, with all of these squirrels having been through torpor–arousal bouts prior to euthanasia by decapitation and tissue sampling, as previously described [23]. Tissue samples were shipped to Carleton University on dry ice and then stored at −80 °C until use.

### 2.2. Total Protein Extraction for Immunoblots

Total protein was extracted from squirrel brown adipose tissue from the control and late torpor samples (*n* = 6 independent biological replicates obtained from the same cage). The samples were mixed with prechilled 1:2 w:v homogenization buffer (20 mM Hepes, pH 7.4, 100 mM NaCl, 0.1 mM EDTA, 10 mM NaF, 1 mM Na_3_VO_4_, 10 mM β-glycerophosphate), with the 1 µL/mL protease inhibitor cocktail (Bioshop, Burlington, ON, Canada, catalog no. PIC001.1) (reconstituted in 100 mL of deionized water) added simultaneously with mM phenylmethylsulfonyl fluoride. Following homogenization and 15 min of incubation on ice, the samples were centrifuged at 14,000× *g* for 20 min at 4 °C. A Bio-Rad protein assay (Bio-Rad, Mississauga, ON, Canada), Bradford assay (Bio-Rad; Cat #5000006), using bovine serum albumin as the standard, was conducted to determine the protein concentrations of the resulting supernatants. All samples were standardized to a constant protein concentration via the addition of small volumes of homogenization buffer. The samples were stored at −80 °C for further use.

Equal volumes of total soluble protein extracts were then mixed with 1:1 *v*/*v* 2× loading buffer (100 mM Tris-HCl, 4% *w*/*v* SDS, 20% *v*/*v* glycerol, 0.2% *w*/*v* bromophenol blue, and 10% *v*/*v* 2-mercaptoethanol). All samples were then boiled and cooled immediately on ice. Samples were stored at −80 °C until use.

### 2.3. SDS PAGE and Immunoblotting

As previously described [24,25], 25 µg of the protein from samples from both conditions—control and late torpor—was loaded into each lane of a 5% upper stacking gel and 8, 10, 12, or 15% SDS PAGE resolving acrylamide gel based on the molecular weight of the protein of interest (acrylamide/bis-acrylamide ratio 29.2:0.8; *w*/*w*) along with a BLUelf Prestained Protein Ladder (Froggabio: PM008-0500-G, FroggaBio Inc., Concord, ON, Canada) in the first lane. Proteins were transferred to polyvinylidene difluoride (PVDF) membranes at a constant current of 160 mA for 90 min after. Membranes were washed with TBST (20 mM Tris, pH 7.5, 150 mM NaCl, 0.05% Tween-20) (3 × 5 min) on a shaking platform. Following blocking with 3% *w*/*v* non-fat milk (1 × 30 min) and washing again (3 × 5 min), membranes were incubated with primary antibody (1:1000 *v*/*v* TBST) on a rocker overnight at 4 °C (Appendix A). Primary antibodies were chosen based on the strong sequence homology of ground squirrel proteins to immunogen sequences using BLAST (version 2.17.0). Subsequently, membranes were incubated with anti-rabbit secondary antibody diluted 1:5000 in TBST for 30 min at room temperature. Bands were visualized by adding hydrogen peroxide and luminol. Detection and quantification were done using a Chemi-Genius Bio-Imaging system and Gene Tools software (Syngene, Baltimore, MD, USA; version 4.3.8.0).

Protein bands were standardized against a Coomassie-stained PVDF membrane for the same protein [26,27]. The analysis was conducted using a Student’s *t*-test (*n* = 6) using RBioplot. *p* < 0.05 was accepted as a significant difference between groups. Full blots are shown in supplementary figures (Figure S1).

## 3. Results

Relative protein levels of major components of poly(A) tail machinery were analyzed in control and late torpor conditions in the brown adipose tissue of the 13-lined ground squirrel using Western blots.

### 3.1. Cleavage and Polyadenylation Complex

**Figure 1:**
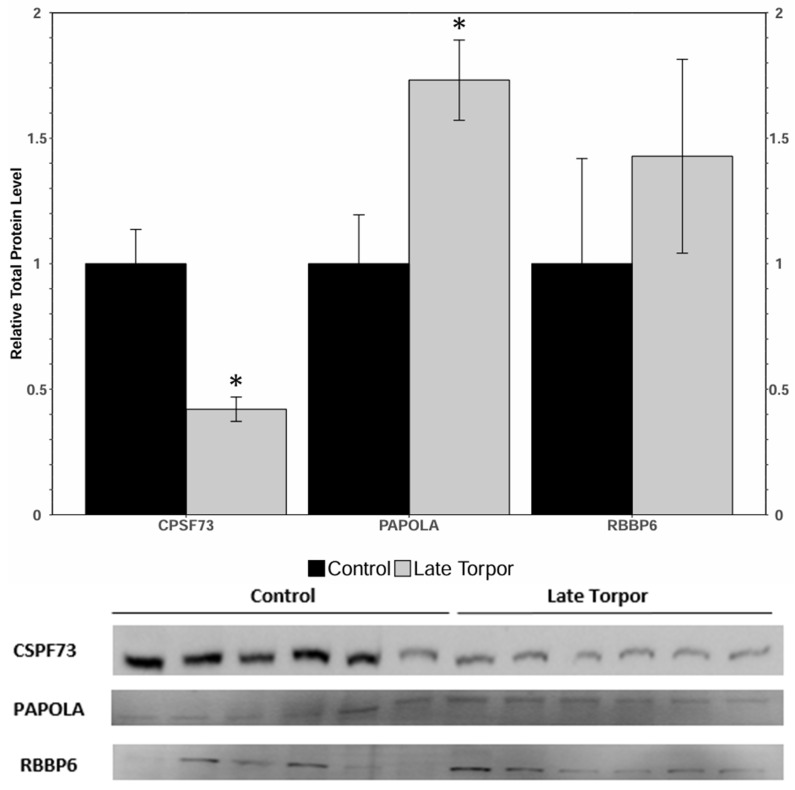
Relative protein expression levels of CPSF73, RBBP6, and PAPOLA in brown adipose tissue of *I. tridecemlineatus* during control and late torpor conditions as determined by Western immunoblotting. Corresponding Western immunoblot bands are shown below histograms. Data are means ± SEM; *n* = 6 independent trials on samples from different animals. Data were analyzed using analysis of variance with a Students *t*-test; asterisks denote values that are significantly different from each other (*p* < 0.05).

Analysis of catalytic and regulatory components of the CPSF complex was conducted (Figure 1). The catalytic sub-unit of the complex, CPSF73, exhibited a significant reduction in protein abundance of 68 ± 5% from control to late torpor in BAT. Poly(A) tail adenylase, PAPOLA, experienced a 1.73 ± 0.16-fold increase, while RBBP6 showed no significant change in protein level (Figure 1).

### 3.2. Poly(A)-Binding Proteins and Translation Initiation

**Figure 2:**
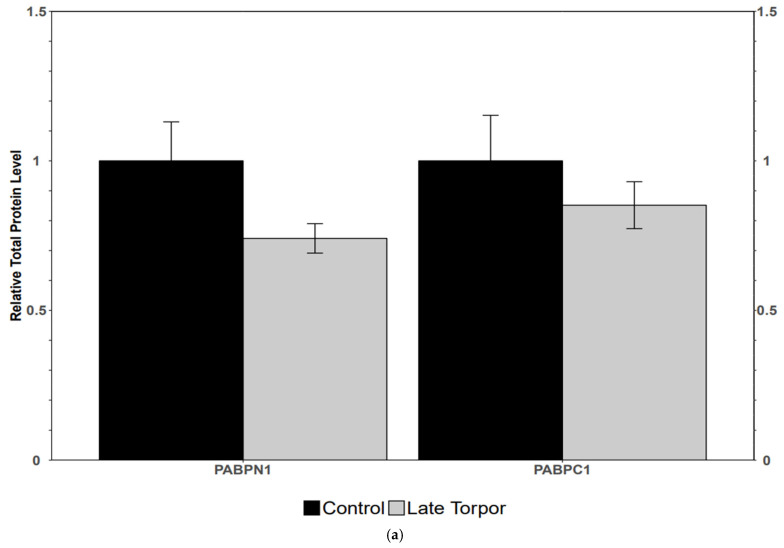

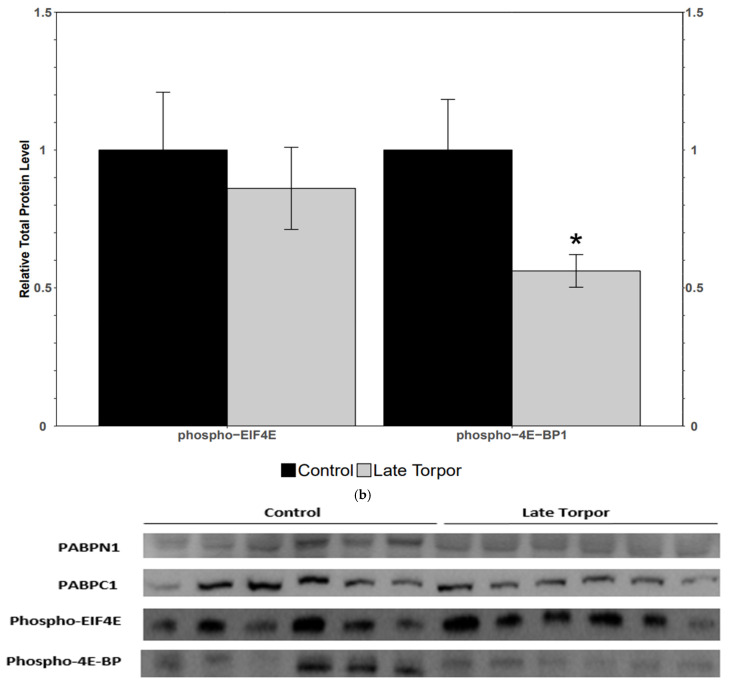
(**a**) Relative protein expression levels of PABPN1 and PABPC1; (**b**) relative protein expression levels of phosphor-eIF4E and phospho-4E. All other information as in Figure 1.

Poly(A) tail binding proteins PABPN1 and PABPC1 showed no significant change in protein levels from control to late torpor in BAT (Figure 2a). Protein level analysis of the cap-dependent PABPC1 binding translation initiator in its active form, phospho-eIF4E, interestingly depicted no significant change. The potent inhibitor of this protein, 4E-BP1, however, saw its phosphorylated variant face a significant reduction in protein abundance of 44 ± 6% during late torpor, implying increased affinity for eIF4E (Figure 2b).

### 3.3. Poly(A) Tail Deadenylation Machinery

**Figure 3:**
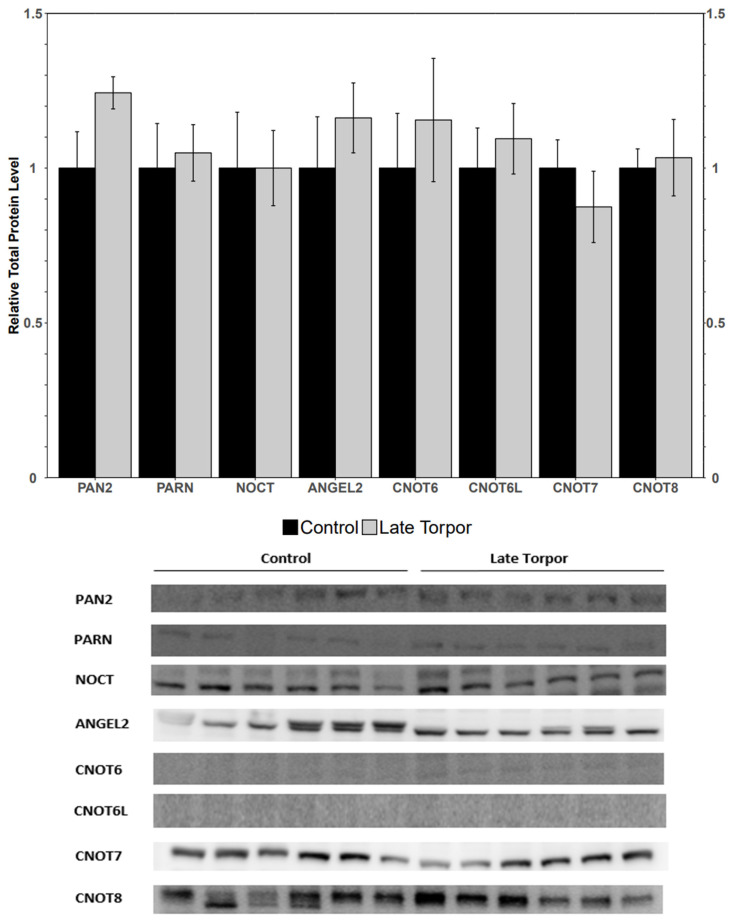
Relative protein expression levels of PAN2, PARN, NOCT, CNOT6, CNOT6L, CNOT7, and CNOT8. All other information as in Figure 1.

Analysis of the protein levels of poly(A) tail deadenylases PAN2, PARN, NOCT, and ANGEL2 in late-torpor BAT depicted no significant change in protein abundance from control to late torpor. Similarly, CCR4-Not proteins CNOT6L and CNOT6-8 showed no significant changes (Figure 3).

## 4. Discussion

Animals that frequently undergo bouts of torpor as a survival strategy must also meet the requirement for rapid reactivation of cellular processes upon arousal. The 13-lined ground squirrel can meet these requirements consistently throughout the cold winter months by efficiently entering a global metabolic rate depression throughout torpor, followed by a rapid reactivation of vital cellular processes upon arousal [2,5,28]. At the cellular level, this survivability is in part a result of regulatory control, often through global suppression of de novo transcription and translation [29,30]. This suppression of vital mechanisms, while maintaining tissue viability across many torpor–arousal cycles, is what makes these organisms ideal models to study stress tolerance. Post-transcriptional control of mRNA fate is one of these vital mechanisms, as metabolic suppression of gene expression during torpor is just as important as its thermogenic activation upon arousal. In recent studies, the poly(A) tail and associated binding proteins have emerged as a central mechanism in mRNA stability and regulation [31]. The poly(A) tail is a stretch of adenosine residues added to the 3′ end of eukaryotic mRNAs, playing a key role in transcript stability, nuclear export, and translational efficiency [32,33]. Depending on its interactions with poly(A)-binding proteins, the poly(A) tail length directly influences mRNA half-life and ribosomal recruitment [34]. The poly(A) tail length is also an important aspect of early development, where transcripts are synthesized in advance and stored in a quiescent state until fertilization occurs [35,36]. Gene regulation in oocytes, for example, is largely determined by poly(A) tail length [37]. This study examines enzymes involved in poly(A) tail regulation as key determinants of mRNA stability, storage, and translational readiness, to better understand the effects of torpor on gene expression in BAT.

Prior to poly(A) tail synthesis, a mature pre-mRNA must be cleaved by CPSF (cleavage and polyadenylation specificity factor) [38]. By binding to the specific AAUAAA sequence in an mRNA, CPSF guides cleavage and recruits poly(A) polymerase alpha (PAPOLA) to catalyze poly(A) tail adenylation [39,40]. CPSF73 is a crucial enzyme in this process as it cleaves the pre-mRNA to produce the mature mRNA set for poly(A) adenylation. This enzyme was one of a few with significant changes in protein abundance under stress, showing a decrease of 68 ± 5% in late torpor relative to active control squirrels (Figure 1). Generally, reduced CPSF73 would suggest a decreased capacity for generating new, mature mRNA [41]. This would be a functional change during torpor, as preventing mRNA translation would be consistent with an overall metabolic rate depression [42]. Interestingly, PAPOLA is significantly upregulated during torpor by 1.73 ± 0.16-fold; it is an enzyme that controls the length and thus the stability of a successfully cleaved mRNA transcript. This result lends support to the idea that during torpor, BAT prioritizes maintenance of existing transcripts, but may limit maturation of new mRNA. It could also suggest that mRNA considered valuable enough to mature during torpor requires exaggerated poly(A) tail adenylation for stability and eventual translation. This strategy would help stabilize select mRNA, perhaps in preparation for periods of arousal, while preventing expensive wholesale gene expression during bouts of torpor. Regulatory components of the CPSF complex, such as RBBP6 (Retinoblastoma-Binding Protein 6) exhibited no change between control and torpor states. The current study thus depicts dynamic protein abundance changes in catalytic components of the CPSF complex (PAPOLA, CPSF73), suggesting a possible functional importance of these changes. The modulation of pre-mRNA appears to serve as a checkpoint, controlling whether transcripts progress to eventual protein synthesis, or remain restrained. This also parallels known developmental strategies that demonstrate fine-tuned control of RNA turnover [43].

Polyadenylation is followed by binding of the poly(A) tail-binding proteins that protect mRNA from decay, while also promoting interactions with translational enzymes such as eIF4E (eukaryotic initiation factor 4E) once in the cytoplasm [44,45]. PABPN1 and PABPC1 showed no significant change in this study, maintaining protein abundance observed in typical active squirrels (Figure 2a). A lack of change in these enzymes suggests preservation of poly(A) tail binding activity, maintaining mRNA protection and stability once mature [46,47]. Past studies postulate that cells under metabolic constraints likely choose to store translation-ready transcripts rather than opt for decay, while also suppressing active translation [11,48]. Interestingly, investigation of the active form of cap-dependent eIF4E showed no significant change, suggesting that translation initiation continues as normal. However, further analysis of the inhibitor of eIF4e, 4E-BP1, showed that the phosphorylated state of this inhibitory enzyme was significantly reduced by 44 ± 6% in late-torpor BAT (Figure 2b). Hypophosphorylated 4E-BP1 has increased affinity for eIF4E, thus increasing inhibition of eIF4E activity. This may lead to a repression of cap-dependent translation, corroborating past studies of similar hypometabolic systems demonstrating limited translation during stress [49].

Poly(A) tail shortening through deadenylation is generally a rate-limiting step in mRNA decay [50]. Deadenylases such as PARN, NOCTURNIN and ANGEL2, and enzymes in the CCR4-NOT complex such as CNOT6, CNOT6L, CNOT7, and CNOT8 contribute to progressive poly(A) shortening, resulting in destabilized transcripts [18,51,52]. This study found that during late torpor, none of the studied deadenylases exhibited significant protein abundance changes (Figure 3). The lack of an increase in deadenylase machinery protein abundance suggests that mRNA turnover is not accelerated during torpor but may still maintain an influence over mRNA regulation. This state of maintained deadenylase levels, along with the upregulation of poly(A) tail growth protein PAPOLA, parallels ongoing poly(A) tail growth and removal seen in oocytes until developmental progression resumes [37]. It is thus possible that a reversible state of hypometabolism requires ongoing poly(A) tail length control.

## 5. Conclusions

Taken together, the poly(A) tail regulatory landscape in brown adipose tissue during torpor reflects a highly controlled post-transcriptional state in which transcript production is limited, transcript stability is preserved, and translation is reversibly suppressed. Downregulation of pre-mRNA cleavage enzyme CPSF73 coupled with upregulation of poly(A) tail adenylase PAPOLA suggests a maintenance of existing or vital-to-survival mRNA transcripts, while preventing maturation of typical active state genes. Unchanged poly(A)-binding proteins and deadenylases depict a framework remaining in the steady state that is poised for arousal while not actively committing mRNA to decay throughout the torpid state. Downstream translational repression via 4E-BP1-mediated inhibition of eIF4E further corroborates a lack of commitment to actively translating transcripts in a metabolically suppressed state. This model depicting efficient bottlenecks in rate-limiting steps of the mRNA-to-protein pathway is well suited to the physiological role of brown adipose tissue. It demonstrates that the tissue is translationally restrained while maintaining the ability to reactivate rapidly upon arousal. These findings highlight the importance of poly(A) tail regulation as a key component of reversible cellular suppression and reactivation and its broader relevance to stress tolerance. Future studies should explore if these compelling changes to poly(A) tail regulatory machinery are reflected in poly(A) tail length distributions in BAT during torpor.

## Data Availability

The data that supports the findings of this study are available from the corresponding author upon reasonable request.

## Supplementary Materials

The following supporting information can be downloaded at: https://www.mdpi.com/article/10.3390/jdb14020021/s1. Figure S1: Western blot Images.

## Abbreviations

The following abbreviations are used in this manuscript:

13LGS	13-lined ground squirrel
BAT	Brown adipose tissue
Poly(A)	Polyadenylation
PAPOLA	Poly(A) polymerase alpha
PABP	Poly(A) Binding Protein
CPSF	Cleavage and polyadenylation specificity factor
CCR4	Carbon catabolite repression 4
PAN	Poly(A) nuclease deadenylation complex
NOCT	Nocturnin
PARN	Poly(A)-specific ribonuclease

## Appendix A

**Table A1:** Summary of primary antibody information.

Antibody	Company	Catalog Number
CPSF73	Abclonal (Woburn, MA, USA)	A2222
RBBP6	Abclonal	A14776
PAPOLA	Invitrogen (Carlsbad, CA, USA)	PA5-30414
PABPN1	Invitrogen	PA5-102924
PABPC1	Invitrogen	PA5-66900
Phospho-eIF4E (ser209)	Cell Signalling (Danvers, MA, USA)	9741
Phospho-4E-BP (ser65)	Cell Signalling	9451
PARN	Genetex (Hsinchu, Taiwan)	GTX115020
PAN2	Abclonal	A15373
NOCT	Abclonal	A15805
ANGEL2	Abclonal	A12799
CNOT6L	Novus Biologicals (Centennial, CO, USA)	NBP1-84186
CNOT6	Novus Biologicals	NBP1-86564
CNOT7	Abclonal	A8959
CNOT8	Novus Biologicals	NBP2-15930
