# Supplementary material for: Torpor-Induced Regulation of Poly(A) Tail Machinery in 13-Lined Ground Squirrel Brown Adipose Tissue
Source: J Dev Biol. 2026 May 14;14(2):21. doi: 10.3390/jdb14020021 (PMC13214660; doi:10.3390/jdb14020021)
Supplement: Supplementary file 1 [file jdb-14-00021-s001.zip › jdb-4156343-supplementary.pdf]

Western blot Images

A red line is used to represent the band of interest

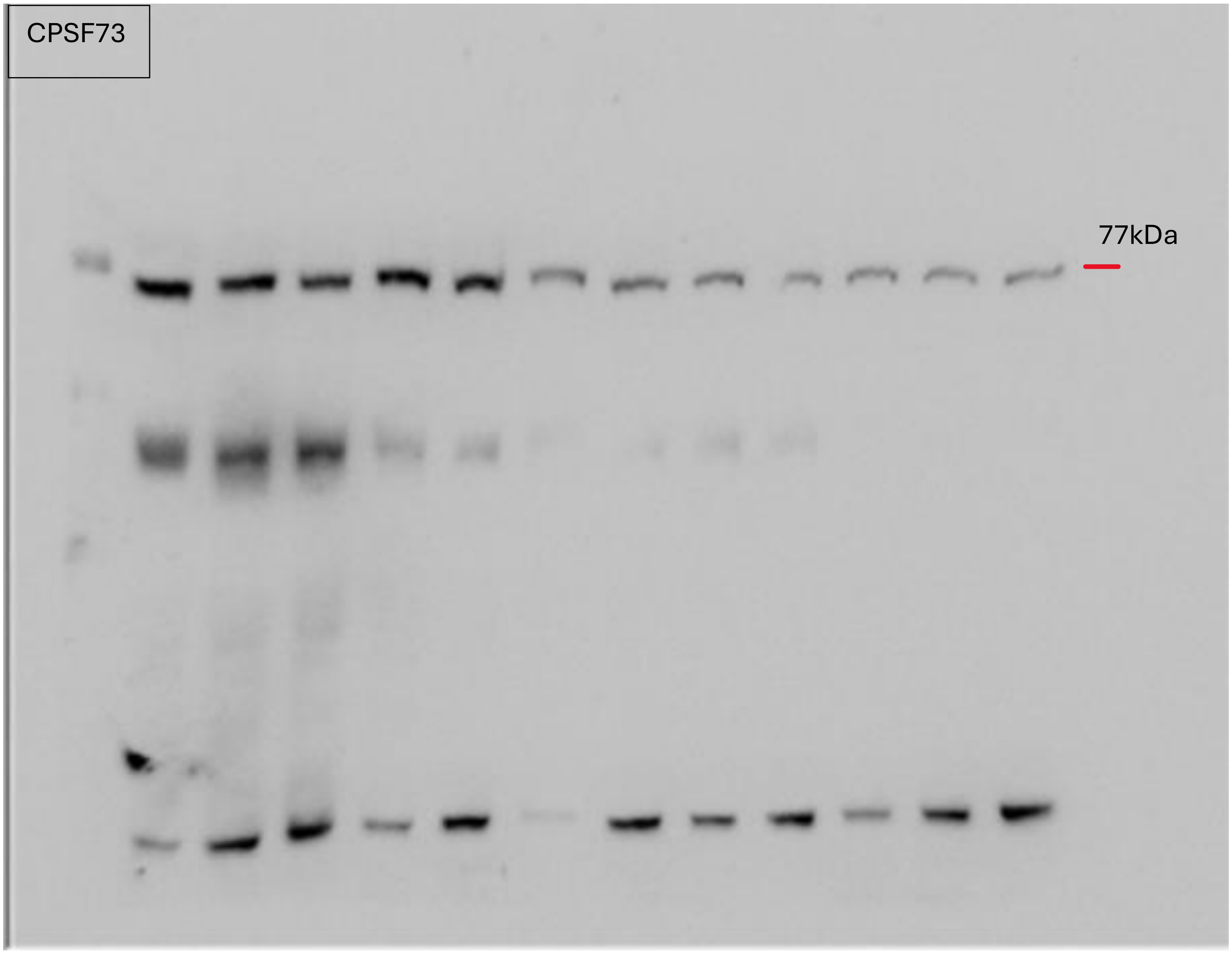

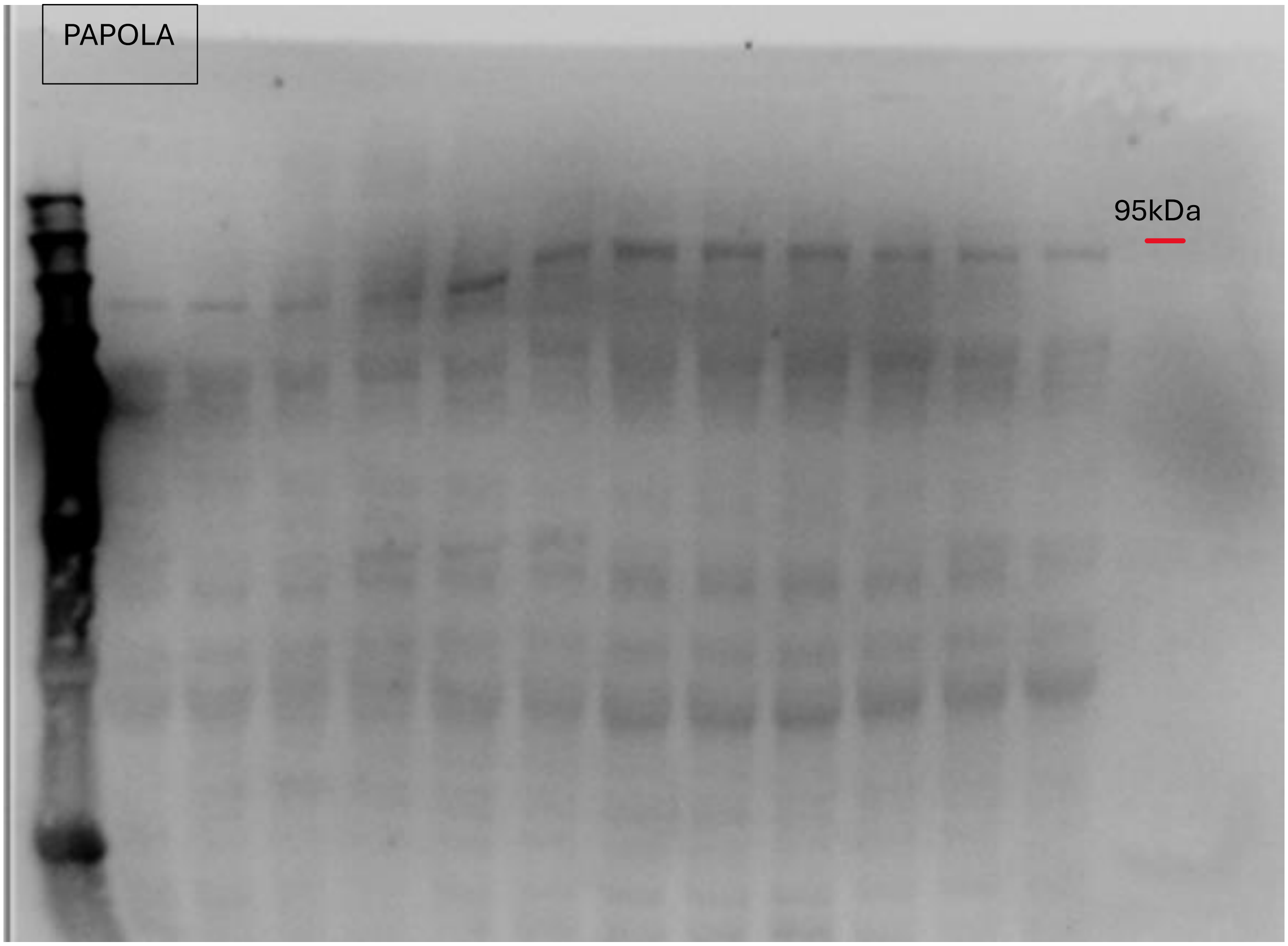
PAPOLA
95kDa

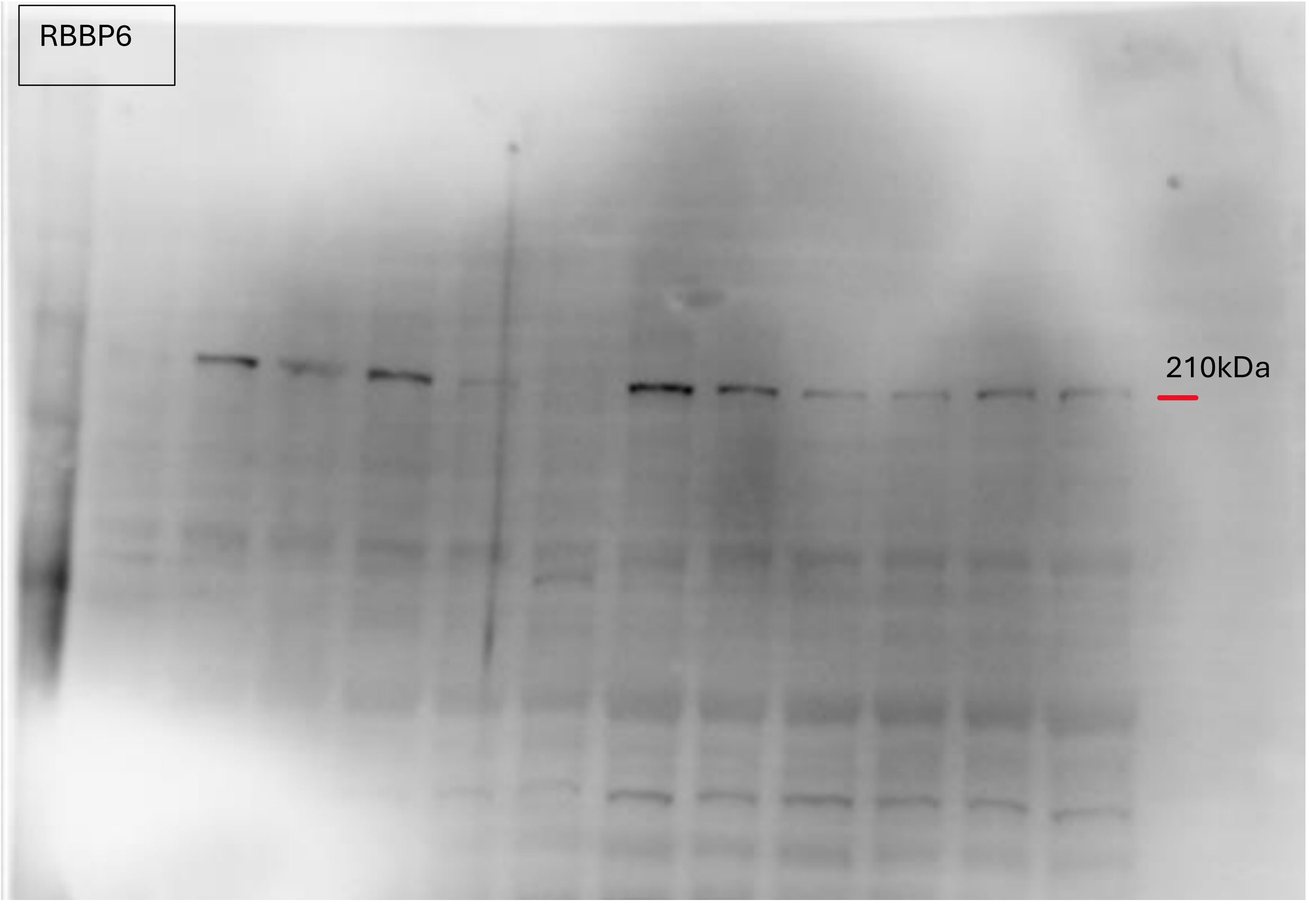
RBBP6
210kDa

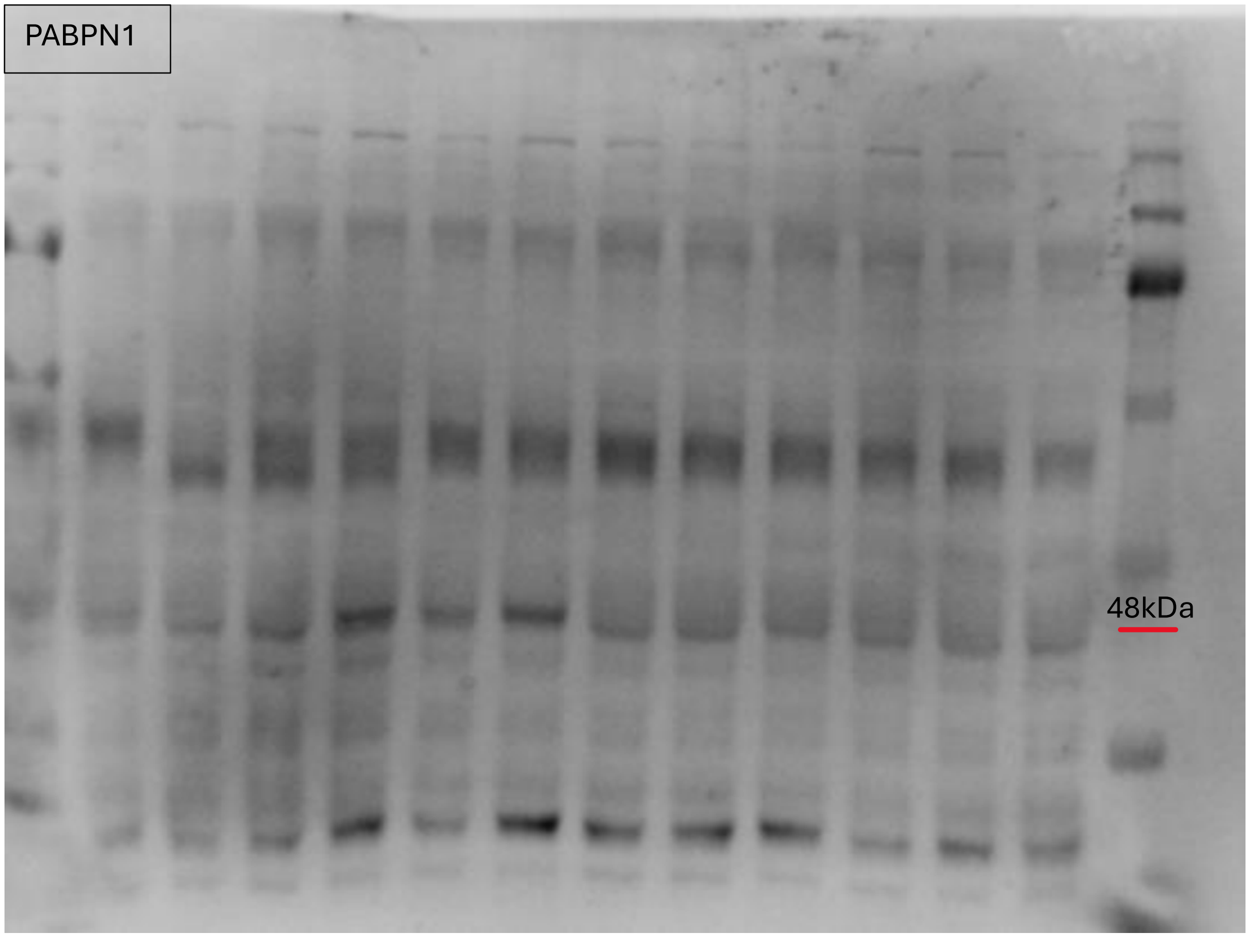
PABPN1
48kDa

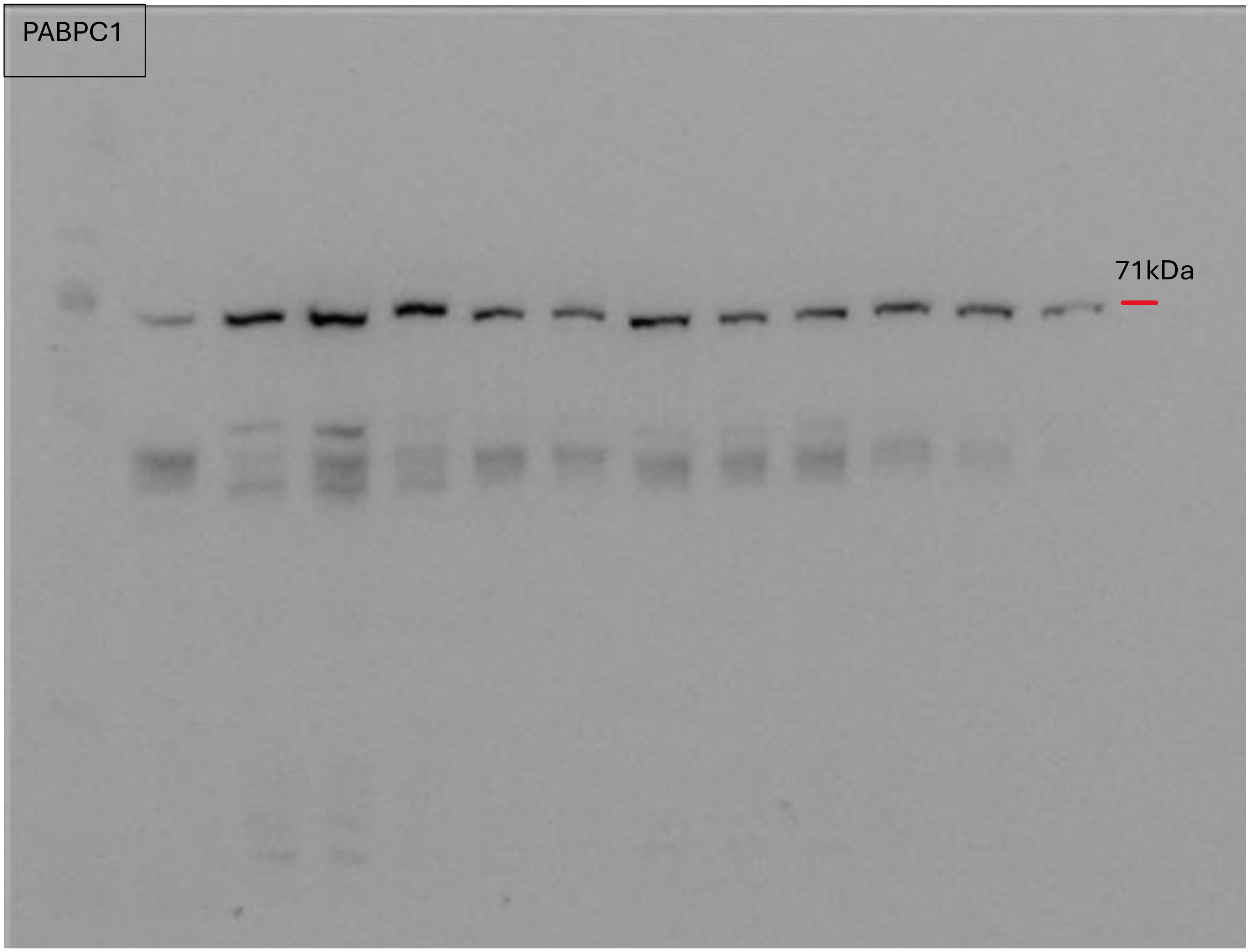
PABPC1
71kDa

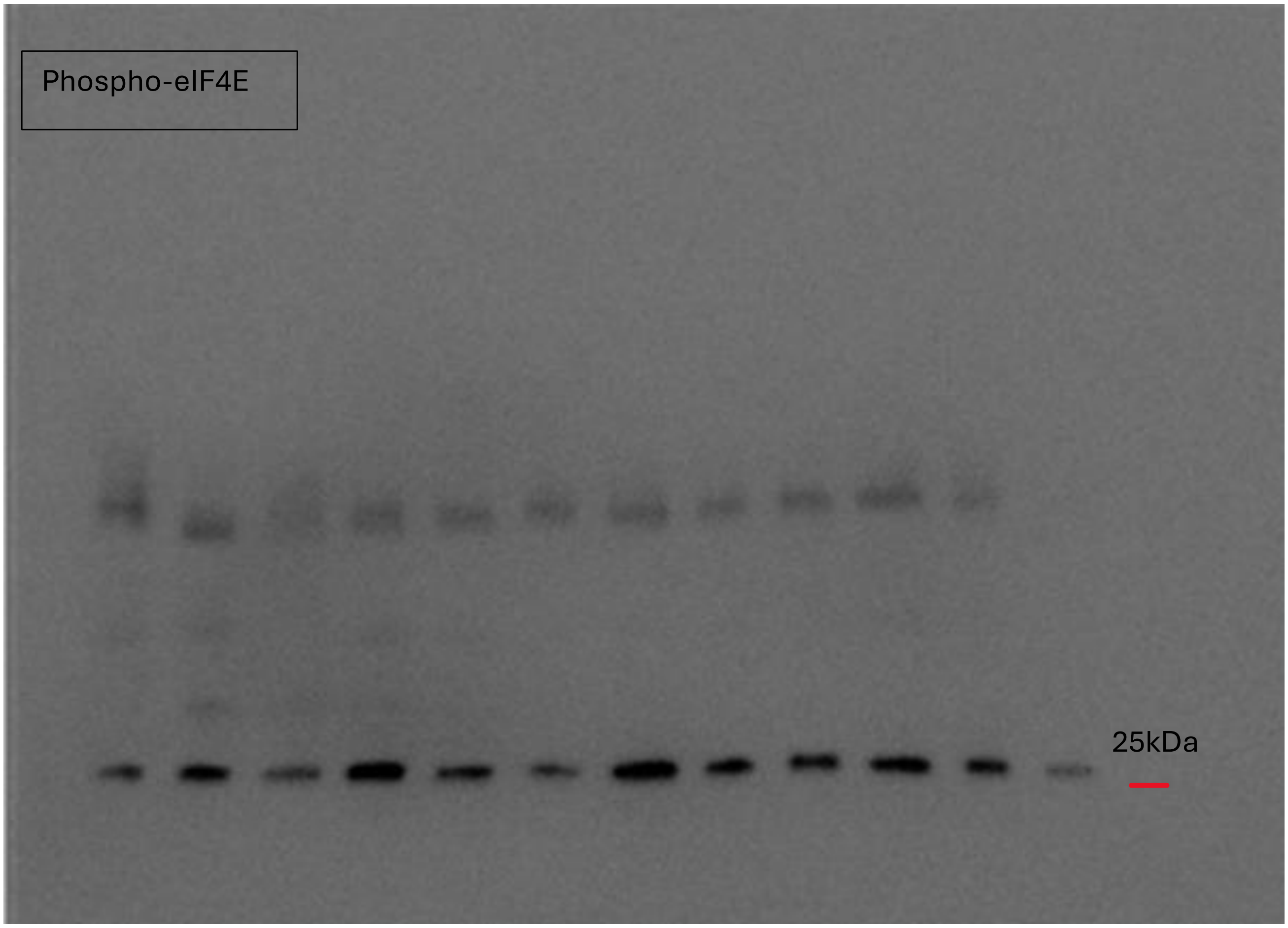
Phospho-eIF4E
25kDa

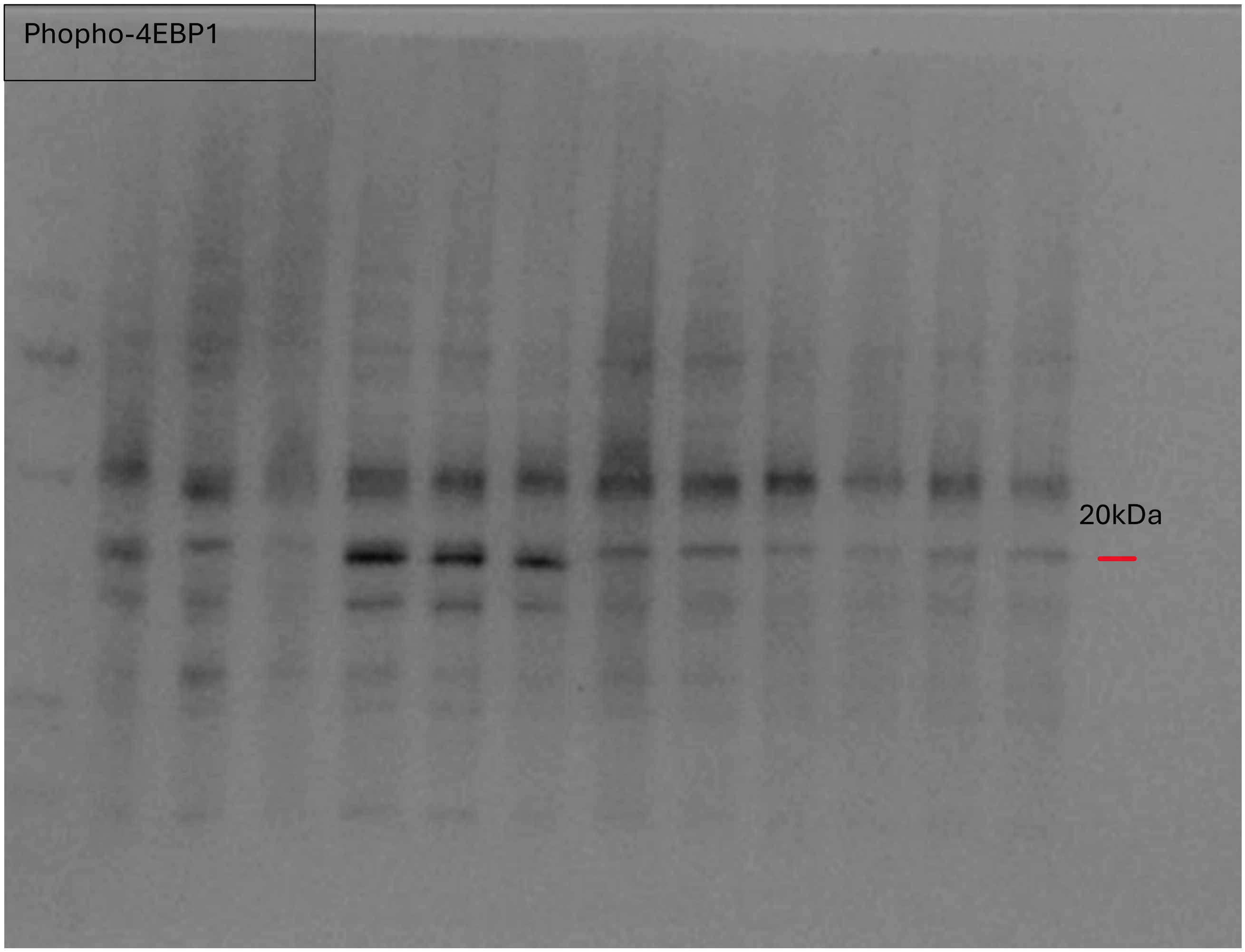
Phopho-4EBP1
20kDa

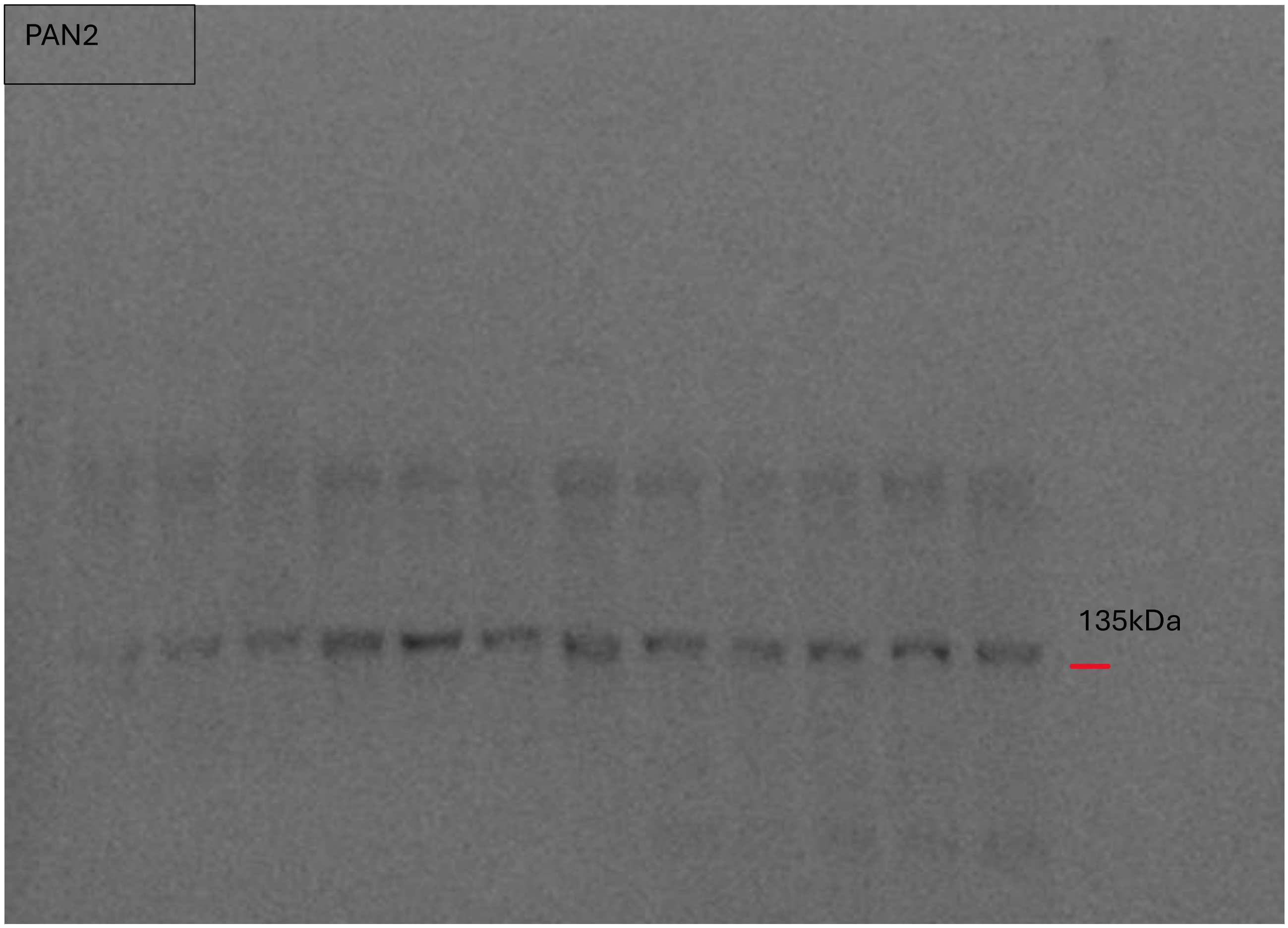
PAN2
135kDa

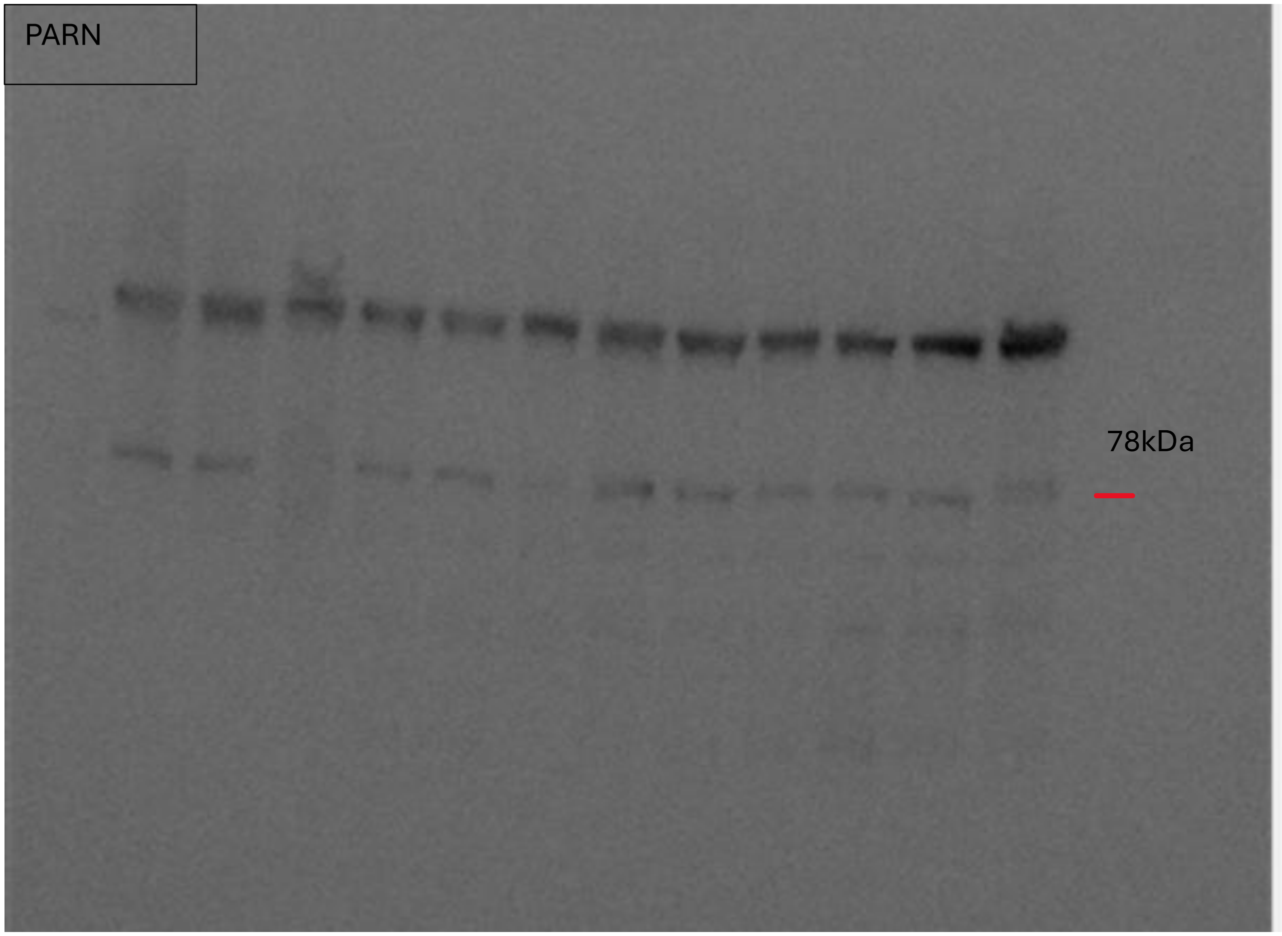
PARN
78kDa

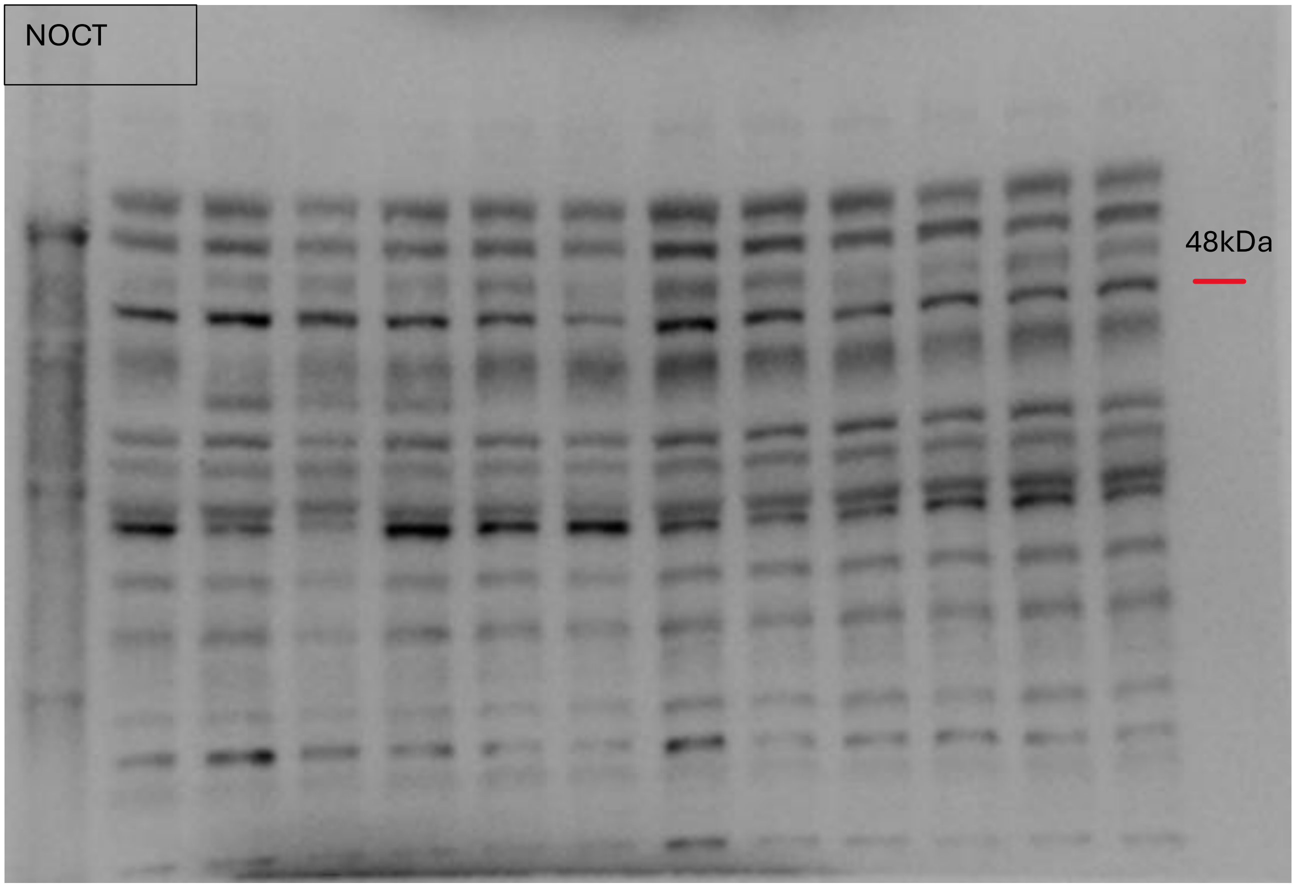
NOCT
48kDa

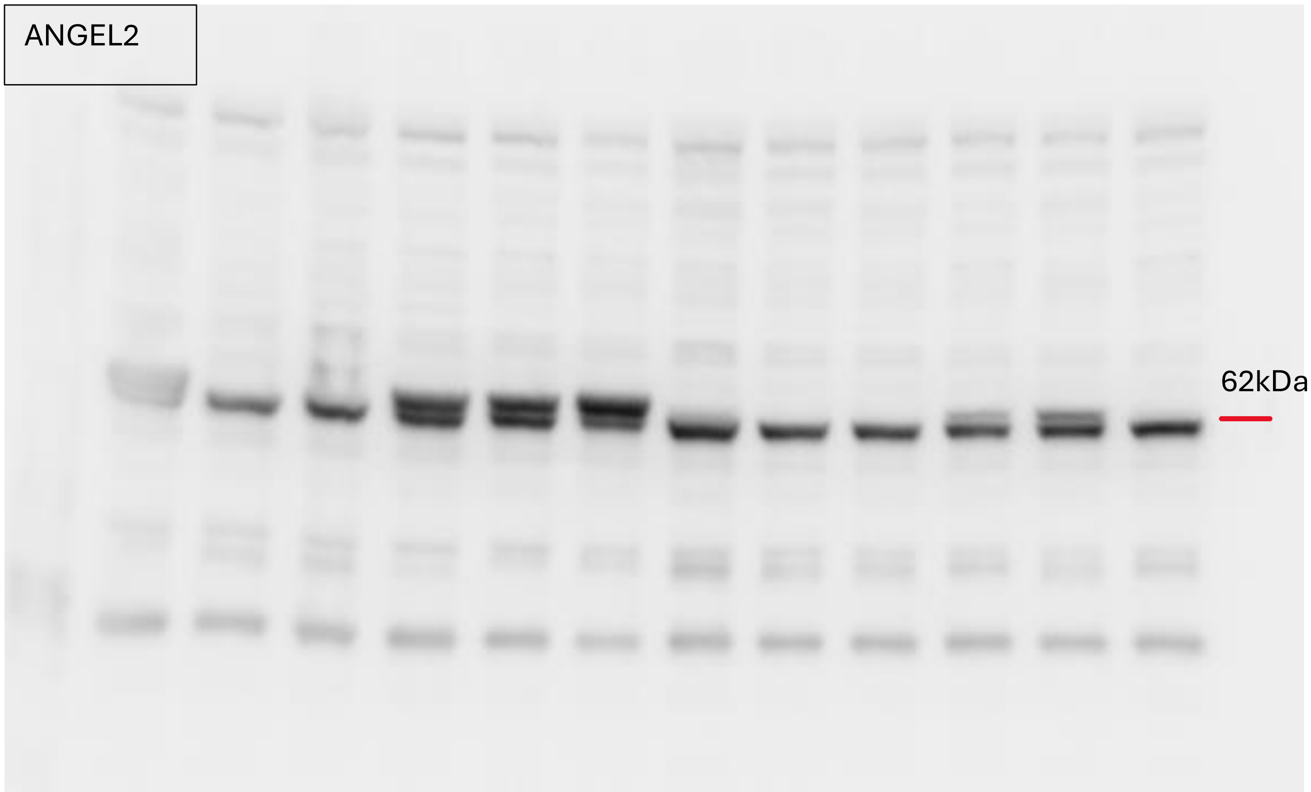
ANGEL2
62kDa

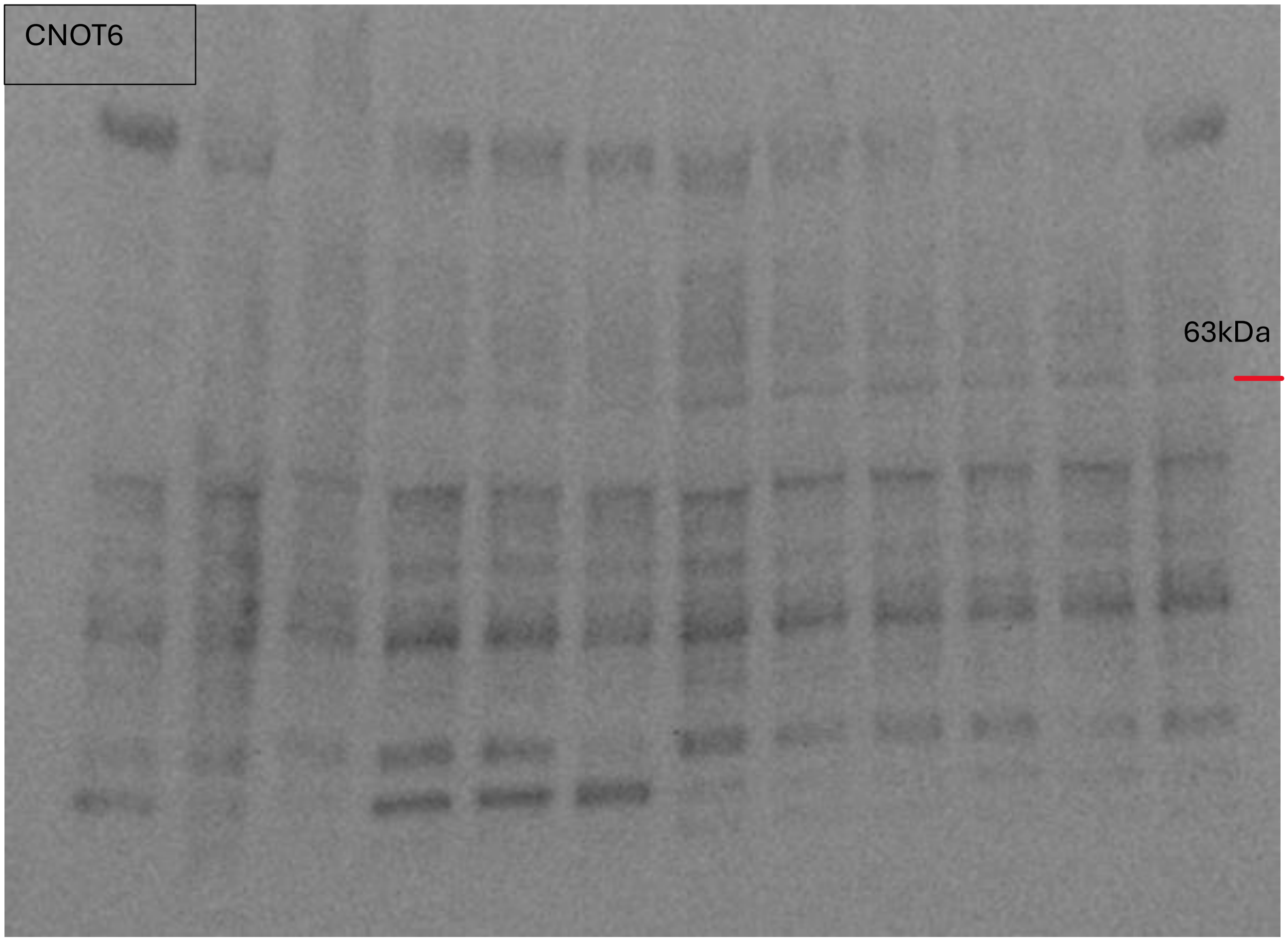
CNOT6
63kDa

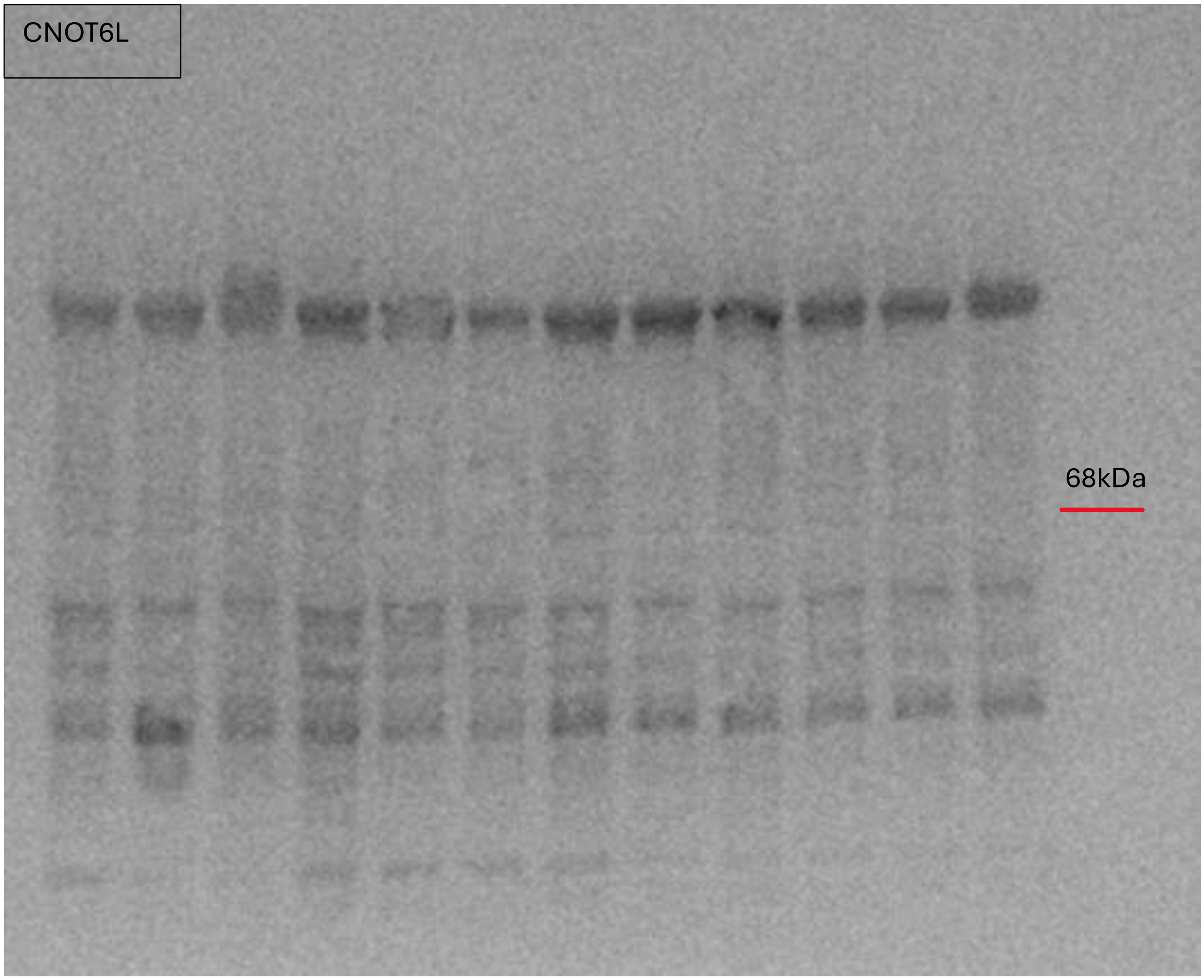
CNOT6L
68kDa

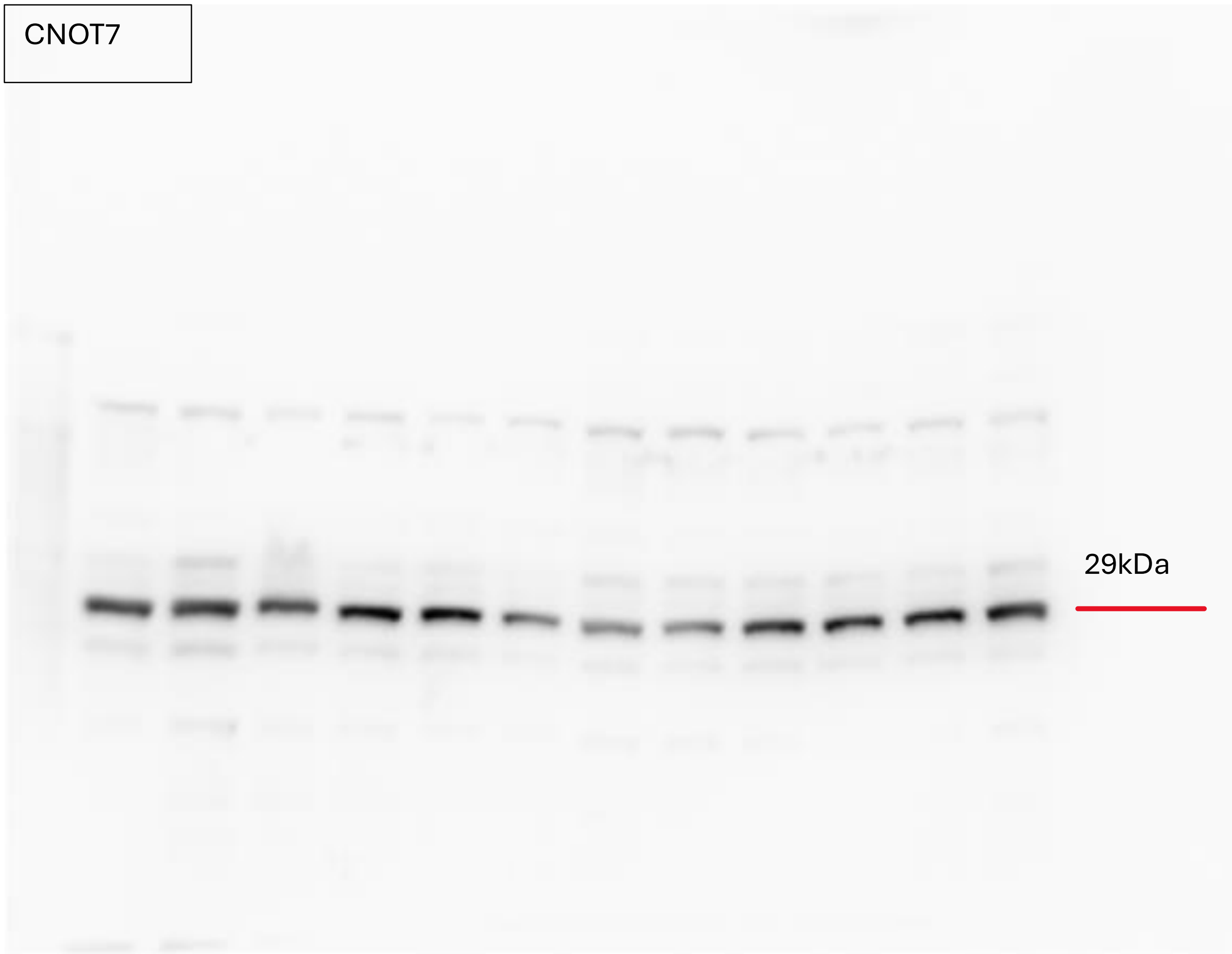
CNOT7
29kDa

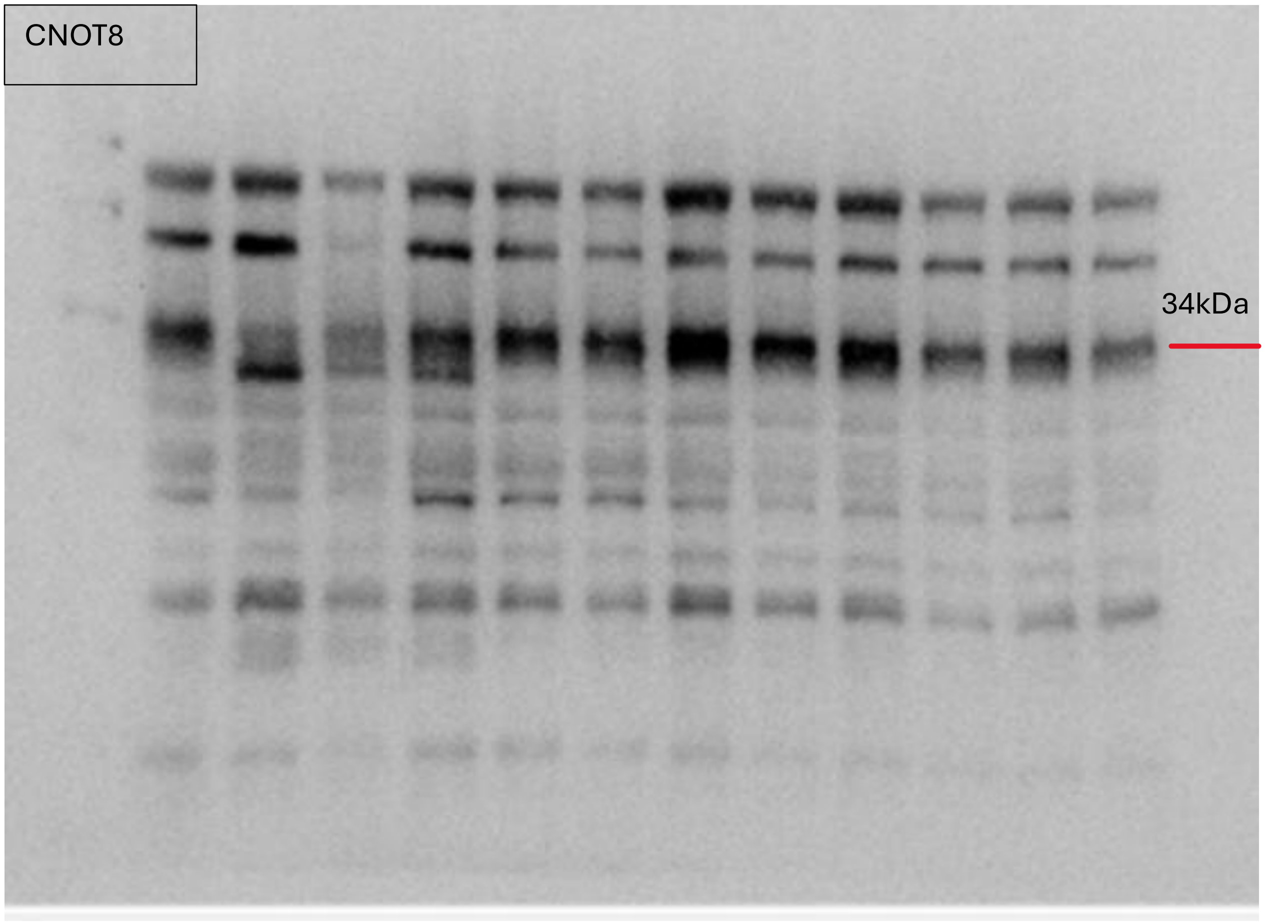
CNOT8
34kDa

Representative Coomasie stain blot Images

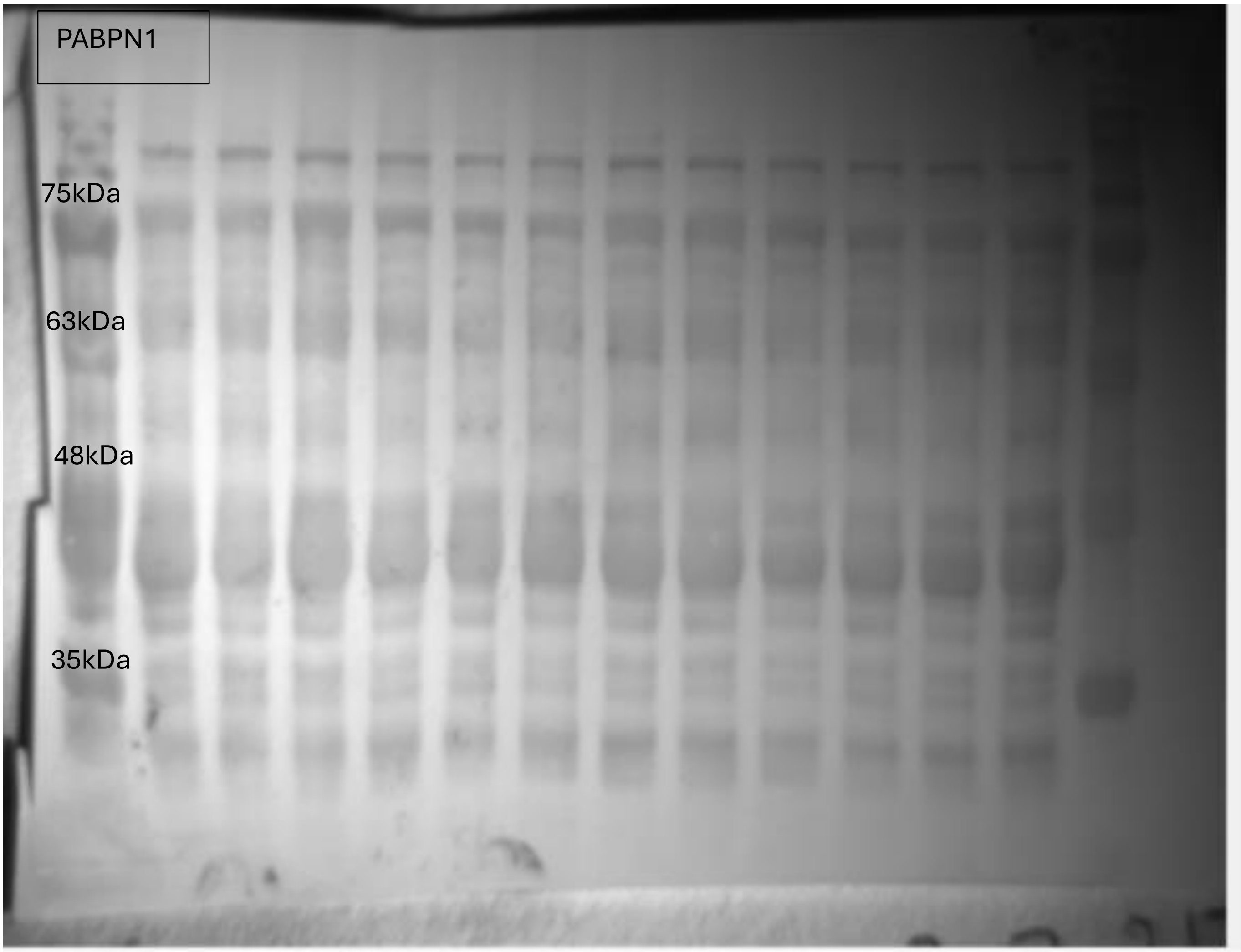

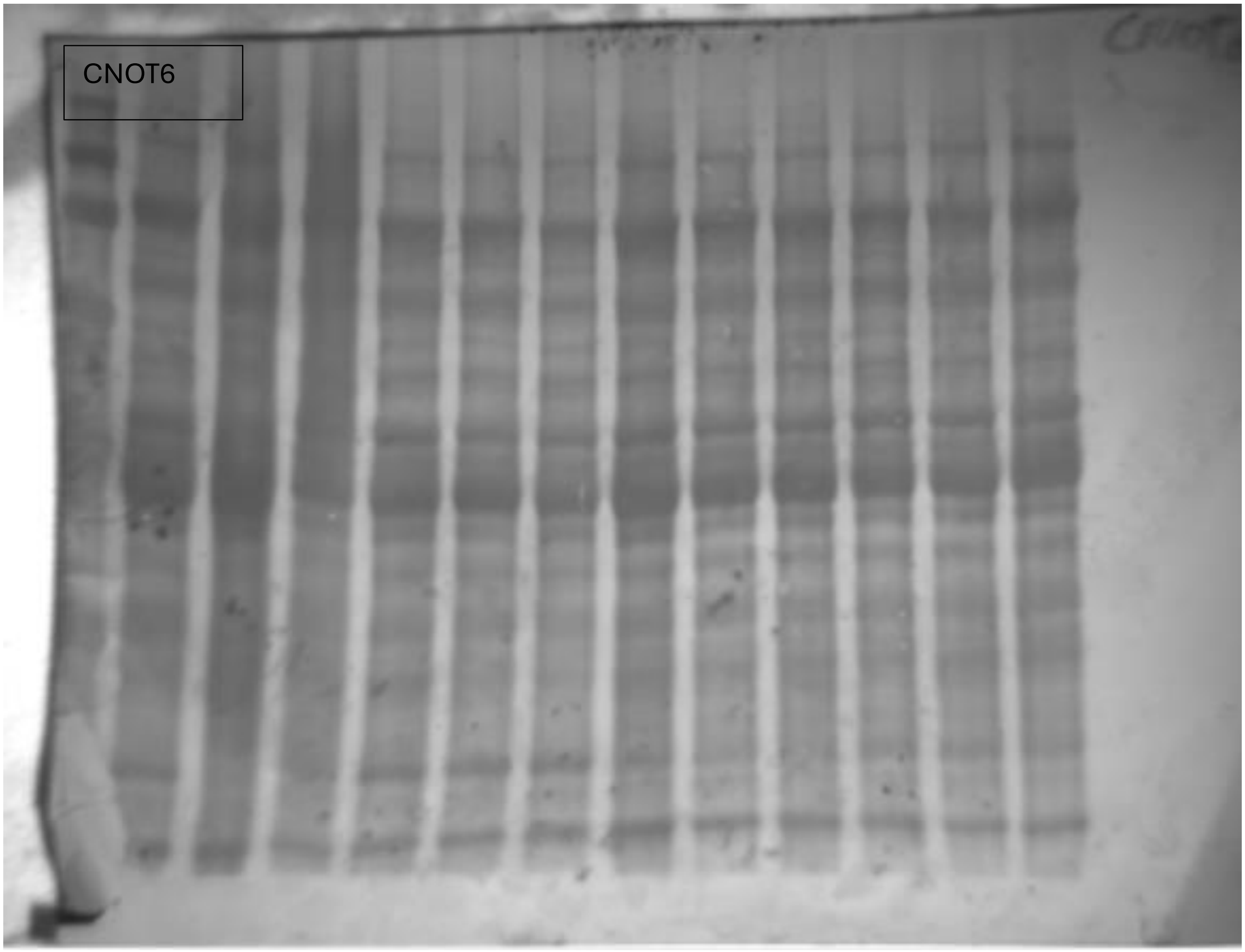
CNOT6
